# Transverse Plane Tendon and Median Nerve Motion in the Carpal Tunnel: Ultrasound Comparison of Carpal Tunnel Syndrome Patients and Healthy Volunteers

**DOI:** 10.1371/journal.pone.0037081

**Published:** 2012-05-11

**Authors:** Margriet H. M. van Doesburg, Jacqueline Henderson, Aebele B. Mink van der Molen, Kai-Nan An, Peter C. Amadio

**Affiliations:** 1 Orthopedic Biomechanics Laboratory, Division of Orthopedic Research, Mayo Clinic, Rochester, Minnesota, United States of America; 2 Department of Plastic, Reconstructive and Hand Surgery, University Medical Center, Utrecht, The Netherlands; Creighton University, United States of America

## Abstract

**Background:**

The median nerve and flexor tendons are known to translate transversely in the carpal tunnel. The purpose of this study was to investigate these motions in differential finger motion using ultrasound, and to compare them in healthy people and carpal tunnel syndrome patients.

**Methods:**

Transverse ultrasounds clips were taken during fist, index finger, middle finger and thumb flexion in 29 healthy normal subjects and 29 CTS patients. Displacement in palmar-dorsal and radial-ulnar direction was calculated using Analyze software. Additionally, the distance between the median nerve and the tendons was calculated.

**Results:**

We found a changed motion pattern of the median nerve in middle finger, index finger and thumb motion between normal subjects and CTS patients (p<0.05). Also, we found a changed motion direction in CTS patients of the FDS III tendon in fist and middle finger motion, and of the FDS II and flexor pollicis longus tendon in index finger and thumb motion, respectively (p<0.05). The distance between the median nerve and the FDS II or FPL tendon is significantly greater in patients than in healthy volunteers for index finger and thumb motion, respectively (p<0.05).

**Conclusion:**

Our results suggest a changed motion pattern of the median nerve and several tendons in carpal tunnel syndrome patients compared to normal subjects. Such motion patterns may be useful in distinguishing affected from unaffected individuals, and in studies of the pathomechanics of carpal tunnel syndrome.

## Introduction

Carpal tunnel syndrome is a peripheral compression neuropathy for which several potential pathophysiological explanations have been proposed. Some studies focus on fibrosis of the subsynovial connective tissue (SSCT) as a cause [1], [2], [3], while other studies focus on dynamic causes, such as a changed motion pattern of the median nerve [4], [5]. Of course, it is possible that the two may be interrelated, in that the fibrosis may affect the motion. Ettema et al. showed that the gliding characteristics in CTS patients are altered, while Osamura et al. showed that the material properties are changed in patients as well [6], [7]. They suggest that these changes may be due to fibrosis of the subsynovial connective tissue and that alterations in the gliding characteristics of the SSCT may affect tendon gliding motion [6].

Even though tendon displacement has been studied before, not much is known yet about the tendon rearrangements within the carpal tunnel with differential finger motion. A pilot study from our institution showed that in index finger and thumb flexion, the motion direction of the median nerve and flexor tendons differs between healthy normal subjects and carpal tunnel syndrome patients, and that it is possible to display these motions with high frequency ultrasound [8]. A change in the biomechanics in the carpal tunnel may be another clue towards identifying the etiology of idiopathic carpal tunnel syndrome, and better insight in the movement of the tendons and the median nerve in the carpal tunnel may assist in designing rehabilitation protocols after surgery.

Ultrasound techniques have been used to examine median nerve and tendon motions in the past [4], [9], [10]. The median nerve is known to move longitudinally within the carpal tunnel, and studies have shown that both the median nerve and the tendons have greater longitudinal excursion in healthy wrists than in symptomatic wrists [4], [5], [10]. The median nerve can also slide transversely within the carpal tunnel and responds to these forces by becoming interposed in various positions between the superficial flexor tendons [4], [11].

In this study, we hypothesized that the motion direction and the displacement of the median nerve and the flexor tendons during differential finger flexion and extension will be altered in CTS patients compared to healthy controls.

## Methods

### Ethics Statement

This research has been approved by the Mayo Clinic Institutional Review Board. We obtained written informed consent from all participants in the study.

### Image Acquisition

**Figure 1:**
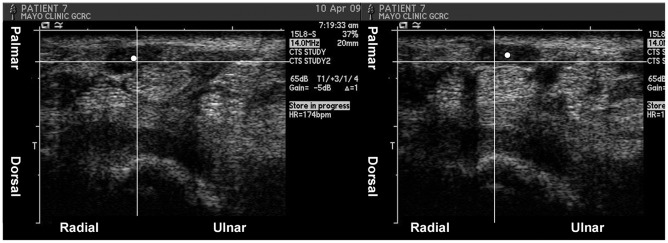
Example of median nerve motion direction measurement in middle finger motion in a patient. The centroid of the median nerve (white dot) was taken in extension (left picture) and flexion (right picture) to calculate motion direction. The grid shows the change in position of the median nerve centroid in ulnar-palmar direction.

After acquiring approval from our Institutional Review Board, we recruited 29 healthy volunteers (15 women, 14 men, age range 22–67 with a mean age of 35.5 years) without any history of CTS, and 29 volunteers with idiopathic CTS (18 women, 11 men, mean age 51.1 years with a range of 26–70 years) which was clinically diagnosed and confirmed by electromyography. All but two volunteers had bilateral CTS. CTS patients were excluded if their medical records showed a history of systemic disease associated with a higher incidence of carpal tunnel syndrome, such as thyroid disease, obesity, rheumatoid arthritis, or any trauma or surgery of the lower arm. The preliminary results from some of the normal subjects in our study population have been published before [8]. In this paper however, we describe the results of the total population compared to CTS patients. Transverse images of the carpal tunnel were obtained using a Siemens Sequoia C512 ultrasound machine (Siemens Medical Solutions, Malvern, PA), with a 15L8 linear array transducer set to a 15 MHz acquisition frequency, placed transversely at the wrist crease and perpendicular to the long axes of the forearm. After obtaining a clear image, the transducer was fixed at its position in a custom made fixture. The depth was set to 20 mm, focus was adjusted to the level of the tendon. The frame rate was set to 30 Hz. The participants were lying down with their hand supinated and strapped to a custom made device, with the wrist in neutral position. Participants were asked to flex and extend their middle finger, index finger and thumb independently from full extension to flexion, until the finger tip touched the hand palm. Also, they were asked to flex four fingers at the same time (index, middle, ring, little finger). In the case of single digit motion, the participant was asked to keep the other fingers as much extended as possible. For all four motions, five cycles of were recorded. Images were reviewed using Analyze 8.1 software (Mayo Clinic, Rochester, MN), selecting the initial and final frames of the motion cycle. The outer hypoechogenic rim of the median nerve and the outer hyperechogenic rim of the tendons were outlined for both the full extension and the full flexion positions. Depending on which motion investigated, we choose to outline the FDS II tendon in case of index finger motion, while in fist and middle finger motion the FDS III tendon and in thumb motion the flexor pollicis longus tendon were measured. Total displacement in both X and Y direction of the tendon and the nerve, the distance between the tendon and the median nerve in flexion and extension, as well as the motion direction of the tendon and the nerve could be calculated, using the centroid of the outlined tendon and nerve (Figure 1). The displacement was defined as the difference in the midpoint coordinates between the extension and flexion position. The intra-rater reliability was calculated from the five cycles that were of each finger motion within each participant. All participants gave written informed consent for this study.

### Statistical Analysis

All results were expressed in mean +/− standard deviation (SD), and all statistical analyses were performed by SAS version 9.2 software (SAS institute Inc., Cary, NC). We used SAS procedure MIXED model approach for statistical analyses, since we evaluated flexion and extension in both left and right wrist of all participants. Participants were treated as repeated factor, wrists (left and right) as random effect factor, and fingers (4 fingers, middle finger, index finger and thumb) or motion direction (flexion/extension) as fixed effect factor. An overall p-value of less than 0.05 was considered significant for finger and motion differences.

## Results

**Figure 2:**
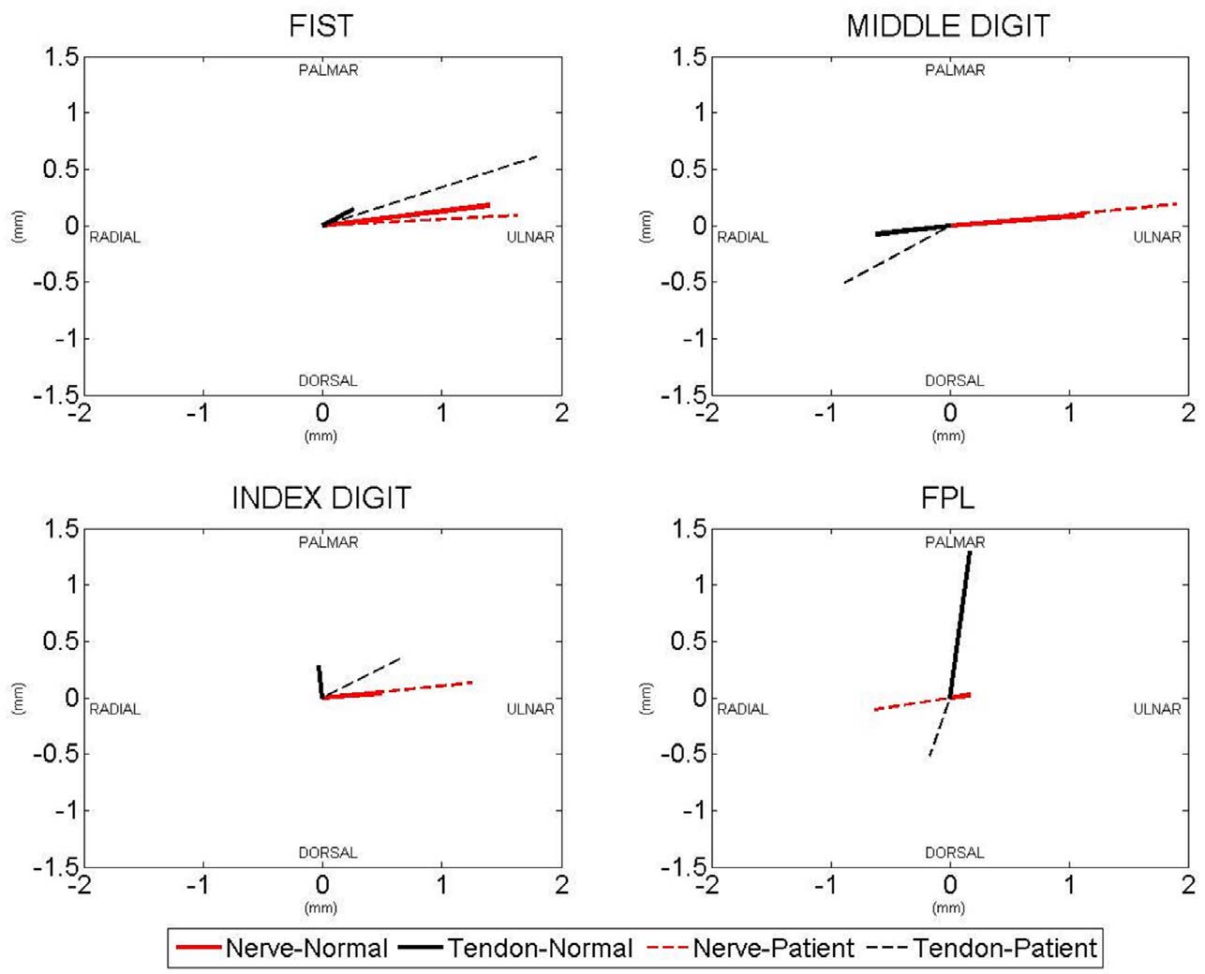
Representation of flexor tendon and median nerve motion direction in CTS patients and normal subjects. For fist and middle finger motion the FDS III tendon was measured, for index finger motion the FDS II tendon and for thumb motion the FPL tendon.

The results are summarized in Table 1 and 2, and in Figure 2.

**Table 1:** Motion of the median nerve, flexor digitorum superficialis tendons and the flexor pollicis longus tendon.

		Ulnar (+) or Radial (−) Motion of Nerve Mean (SD)	Palmar (+) or Dorsal (−) Motion of Nerve Mean(SD)	Ulnar (+) or Radial (−) Motion of Tendon Mean (SD)	Palmar (+) or Dorsal (−) Motion of Tendon Mean (SD)
Fist Motion	Control	1.40 (1.95)	0.18 (0.39)	0.26 (2.28)	0.15 (0.83)
	Patient	1.63 (2.29)	0.09 (0.39)	1.79 (2.73)*	0.61 (1.01)*
Middle Finger Motion	Control	1.13 (2.13)	0.09 (0.38)	−0.62 (1.23)	−0.07 (0.69)
	Patient	1.90 (1.64)*	0.19 (0.33)	−0.88 (1.41)*	−0.50 (0.82)*
Index Finger Motion	Control	0.49 (1.61)	0.04 (0.35)	−0.03 (2.35)	0.28 (1.00)
	Patient	1.25 (1.43)*	0.13 (0.31)	0.68 (1.55)*	0.36 (1.13)
Thumb Motion	Control	0.17 (0.84)	0.02 (0.23)	0.17 (0.99)	1.30 (0.61)
	Patient	−0.63 (0.76)*	−0.10 (0.21)*	−0.17 (0.70)*	−0.51 (0.55)*

For fist and middle finger motion the FDS III tendon was measured, for index finger motion the FDS II tendon and for thumb motion the FPL tendon. Measurements in millimeter (mm).

* p<0.05 between controls and patients.

### Motion Direction

As shown in Table 1 and Figure 2, for four finger motion, there was no difference in median nerve motion direction between normal subjects and patients. The FDS III tendon however, moved more towards the ulnar and palmar side in patients than in normal subjects (p = 0.008 and p = 0.0008 respectively). In middle finger motion the median nerve moved more ulnarly in patients than it did in normal subjects (p<0.0001), while the FDS tendon of the middle finger moved more towards the dorsoradial side (p<0.05). In index finger motion both the median nerve and the FDS II tendon moved more ulnarly in patients, while in normal subjects the tendon moved slightly radial (p = 0.038 and p = 0.027 respectively). In thumb motion the median nerve moved dorsoradial in patients, while it moved palmarly and ulnarly in normal subjects (p<0.05). The FPL tendon moved slightly radial and dorsal in patients as well, while it did not in normal subjects.

### Transverse Distance

As shown in Table 2, in middle finger and four finger motion, the FDS III tendon’s total motion was greater in patients than in normal subjects (p = 0.0042 and p<0.0001 respectively), as well as the median nerve total motion in thumb motion (p = 0.0003). In both index finger and thumb motion, the distance between the nerve and the FDS II and FPL tendon respectively, was greater in CTS patients in both extension and flexion, as well as in fist flexion.

**Table 2:** Displacement and distance between median nerve an flexor tendons.

		Total Displacement Nerve Mean (SD)	Total DisplacementTendon Mean (SD)	Distance between Nerve and Tendon in Extension Mean (SD)	Distance between Nerve and Tendon in Flexion Mean (SD)
Fist Motion	Control	1.93 (1.48)	1.90 (1.54)	4.34 (1.45)	4.29 (1.58)
	Patient	2.20 (1.80)	2.79 (1.90)*	4.25 (1.15)	4.99 (1.91)*
Middle Finger Motion	Control	2.00 (1.40)	1.29 (0.95)	4.18 (1.45)	3.75 (1.22)
	Patient	2.15 (1.34)	1.61 (1.04)*	4.11 (1.08)	3.66 (0.89)
Index Finger Motion	Control	1.37 (1.02)	2.08 (1.50)	4.38 (1.33)	4.46 (1.33)
	Patient	1.54 (1.09)	1.72 (1.15)	5.33 (1.13)*	5.56 (1.33)*
Thumb Motion	Control	0.59 (0.63)*	0.90 (0.80)	6.58 (2.29)	6.46 (2.30)
	Patient	0.81 (0.61)	0.92 (0.49)	7.82 (1.91)*	7.69 (2.05)*

Total displacement (mm) of the median nerve and the different tendons, and distance between median nerve and tendon during finger motion. For fist and middle finger motion the FDS III tendon was measured, for index finger motion the FDS II tendon and for thumb motion the FPL tendon.

* p<0.05 between controls and patients.

## Discussion

This study suggests that there is a changed motion pattern of the median nerve and the flexor digitorum superficialis tendons in the carpal tunnel in CTS patients compared to normal subjects. Also, there seems to be a greater change in distance between the median nerve and the tendon for index finger and thumb motion in CTS patients in comparison to healthy controls.

Many studies have focused on longitudinal motion patterns of the tendons and median nerve in the carpal tunnel [4], [5], [10], while only few have focused on transverse plane motion [12], [13], [14]. To our knowledge no studies have investigated the motion patterns during differential finger motion or have distinguished the exact motion direction of the nerve and the tendons. Our results show greater motion in patients than in normal subjects, while some other studies show irregular and small transverse displacement [4], [13]. Nakamichi and Tachibana studied transverse sliding of the median nerve in asymptomatic human cadavers, using ultrasound [12]. They found a mean transverse sliding of 2.1 mm. Ugbolue et al. found in their study of cadaver hands, with simulated active tendon motion, that the transverse displacement of the index finger and middle finger FDS tendon and the median nerve is relatively small compared to the longitudinal motion [13]. They found values ranging 1.4–5.1 mm transverse displacement in the median nerve and 1.9–7.3 mm for the tendons. These measurements were done in cadavers with no history of CTS. The range of their results is comparable to ours, although they do not specify for each motion in which direction the median nerve or FDS tendon moves specifically. Erel et al. examined both CTS patients and healthy controls, and found that the flexor tendons move palmarly and the median nerve moves radially from flexion to extension, with radial translation values having a mean of 0.89 mm in their 17 CTS patients and a mean of 1.55 mm in their 19 normal subjects [4]. Our results show mostly an ulnar translation. However, while we and Erel et al. studied similar subjects, the methods used were quite different. Our measurements were taken from extension to flexion, while Erel et al. measured from flexion to extension. There were several other differences between our study and that of Erel et al, including software, hardware, and image acquisition rate (Erel et al. at 10 frames per second versus ours at a full video rate of 30 frames per second), but the most important difference may be that we measured full fist motion, while Erel et al. held the wrist and interphalangeal joints fixed, and thus only measured the effect of metacarpophalangeal joint motion. This important difference in the motion that was evaluated may well explain the difference in results.

During image acquisition and analysis, we noticed in the ultrasound clips that sometimes the median nerve would move suddenly, to rapidly snap to a new position, while in most other measurements, the median nerve would slide smoothly in the transverse plane. There seemed to be a trend that these particular patients also showed greater displacement results, but we did not do any statistical analysis of this observation because of the small number of patients showing this pattern. This snapping phenomenon could possibly be explained by a hypothesis provided by Ettema et al. previously: in late stages of SSCT fibrosis the tendons sometimes break free from the adherent synovium, and actually increase their motion relative to the synovium [15]. Indeed, Ettema et al. noted on direct surgical observation that the patients appeared to fall into two groups: those whose tendons and synovium were more adherent than normal, and those in whom the tendons were completely unattached to the synovium. They hypothesized that the latter group represented an end stage situation. Both groups of patients could be distinguished from normal hands (cadavers in their study), where the tendon and synovial motion had intermediate values. This may explain the higher displacement results in our CTS patients. It remains hard to determine which occurs first: fibrosis which might cause a changed motion pattern and then CTS, or a different motion pattern which makes a person prone to develop fibrosis and possibly CTS.

The strength of this study is that we describe, for the first time, the specific motion direction of the median nerve and the different tendons in the carpal tunnel during differential finger motion. We also showed that it is possible to investigate these motions with ultrasound, thus, this method and our results may help to understand carpal tunnel biomechanics. Knowing the changes in biomechanics within the carpal tunnel may aid in understanding the pathophysiological process causing compression neuropathies such as carpal tunnel syndrome. In a more clinical setting, better knowledge of the median nerve and tendon motion in the carpal tunnel may also be helpful in identifying patients with motion patterns similar to CTS patients, who do not have neurological symptoms; it might be useful to follow such patients to determine if neurological symptoms develop in the future, or if the motion patterns can be affected by rehabilitation exercises.

One of the weaknesses of our method is that ultrasound is known for great operator dependency and the variability in our results could be caused by this, even though all image acquisition was done by the same investigator. Because all measurements were done by the same investigator, we were not able to measure interobserver reliability. Second, there is a difference in the mean age between the patient and the control group, which may have caused bias in our results. However, CTS is known to be more frequent in women and less common in young people, a trend that is represented in our patient group. It is possible though, that the higher average age in the patient group has caused greater differences in measurements, which could be due to a normal aging process and not so much to the development of CTS. Third, it would be interesting to see if there is any correlation between the displacement of the nerve and the tendons and the severity of electrophysiological studies, this study did not correlate our results to severity of electrophysiological studies. Despite our study not having enough power, preliminary analysis seems to show a trend that the more severe the EMG results are, the lesser the median nerve moves. This would correspond with earlier findings and also with intra-operative observations. Therefore, for future studies it would be interesting to know if there is any correlation between electrophysiological severity and median nerve motion in the carpal tunnel. We did our measurements with the wrist in neutral position. Yoshii et al. showed in a cadaver study that median nerve and tendon motion decrease with wrist flexion. Since they looked at longitudinal motion, it would be interesting to see if wrist position also affects transverse motion of the structures within the carpal tunnel [16].

In conclusion, our results suggest that with index finger and thumb motion, there is more distance between the nerve and the tendon in CTS patients than in normal subjects. This may aid in understanding the biomechanics within the carpal tunnel and further research needs to elucidate if this method may be useful to assess pathological changes within the carpal tunnel.
